# Retinal Vascular Features in Ocular Blunt Trauma by Optical Coherence Tomography Angiography

**DOI:** 10.3390/jcm9103329

**Published:** 2020-10-16

**Authors:** Daniela Montorio, Luca D’Andrea, Gilda Cennamo

**Affiliations:** 1 Department of Neurosciences, Reproductive Sciences and Dentistry, University of Naples “Federico II”, 80131 Naples, Italy; da.montorio@gmail.com (D.M.); dandrea.luca91@gmail.com (L.D.); 2 Public Health Department, Eye Clinic, University of Naples “Federico II”, 8013 Naples, Italy

**Keywords:** OCTA, vessel density, SD-OCT, ocular blunt trauma, commotio retinae

## Abstract

In this prospective study, we analysed the changes in retinal vessel density (VD) using optical coherence tomography angiography (OCTA) in patients with commotio retinae up to 6 months after blunt ocular trauma. We analysed the VD in the superficial capillary plexus (SCP), deep capillary plexus (DCP), radial peripapillary capillary (RPC) and the foveal avascular zone (FAZ) area at 48 h, and 1, 3 and 6 months after the trauma and compared results with those of healthy fellow eyes. We also evaluated the best-corrected visual acuity (BCVA) and the structural, spectral domain (SD)-OCT parameters: ganglion cell complex (GCC) and retinal nerve fibre layer (RNFL). A total of 18 eyes of 18 patients (8 males, 10 females, mean age 49.61 ± 9.2 years) and 18 healthy control eyes were evaluated. GCC and RNFL thicknesses showed a significant trend towards progressively lower values from 1 month and 3 months after the trauma, respectively, compared to healthy eyes (*p* < 0.005). The reduction in SD-OCT parameters reached a plateau at 6 months. Similar behaviour was found in the VD of the SCP and RPC that significantly decreased, starting from 1 and 3 months after the trauma, respectively (*p* < 0.001). At 6 months, the VD values were stable. The DCP presented an initial decrease of VD (*p* < 0.001), and after 1 month, the values statistically increased until the sixth month, reaching values similar to those of the control group. The FAZ area and BCVA did not show statistically significant changes during the follow-up. OCTA provided a detailed and quantitative analysis of early retinal vascular perfusion alterations after commotio retinae, demonstrating that the impairment of the retinal microvasculature and its progressive changes over time occurred even in the absence of compromised visual acuity.

## 1. Introduction

Commotio retinae (also known as Berlin’s oedema) is a condition involving the outer retinal layers following closed globe trauma, characterised clinically by a transient opacity of the retina in the macula or mid-periphery, resulting in reduced vision that can be temporary or permanent [1]. Histopathologically, it is characterised by significant changes in the outer segments of the photoreceptors that can be restored during the follow-up period [2]. The prognosis is generally good with the recovery of vision within 3–4 weeks.

Structural spectral-domain optical coherence tomography (SD-OCT) has revealed the disruption of the photoreceptor layer and retinal pigment epithelium, showing a hyper-reflectivity of the ellipsoid layer [3,4]. The appearance of this band was revealed to be temporary with a variable recovery that depends on the initial condition of disruption of the outer retinal layers. Previous studies have documented, using SD-OCT, the impairment of the ganglion cell complex (GCC) and the retinal nerve fibre layer (RNFL) after blunt ocular trauma, that while potentially reversible, demonstrates significant axonal damage [5,6,7].

OCT angiography (OCTA) is a non-invasive imaging technique that provides en face visualisation of the retinal vascular networks by detecting the blood flow in different retinal layers. This tool allows for detailed analysis of the retinal perfusion damage in macular and papillary regions that may appear even in the absence of retinal structural loss, as also detected in several optic neuropathies [8]. The aim of this prospective study was to assess the vessel density in macular and papillary regions using OCTA in patients with blunt ocular trauma and to monitor changes over time.

## 2. Experimental Section

### 2.1. Subjects

In this prospective study, we enrolled patients with previous ocular blunt trauma, from January to December 2019 at the eye clinic of the University of Naples “Federico II” during ocular ultrasound examination required in high priority by other departments. The patients underwent an ultrasound examination.

The inclusion criteria were the presence of commotio retinae in the posterior pole due to direct or indirect monocular blunt trauma without any orbital blowout or optic canal fracture, evaluated by slit-lamp biomicroscopy and fundus examination, and SD-OCT results that showed a hyper-reflectivity of the ellipsoid zone. The patients enrolled did not present any changes in the anterior segment (absence of corneal oedema or hypoema). Exclusion criteria were hypertension, heart disease, diabetes, previous ocular surgery, congenital eye disease, high myopia (>6 dioptres), current or previous diagnosis of glaucoma, optic disc anomaly, macular or vitreoretinal diseases, presence of significant lens opacification and reduced-quality OCT and OCT-A images.

All patients were evaluated at 48 h, and 1, 3 and 6 months after the ocular trauma. Each affected eye was compared with their healthy contralateral eye (18 eyes) that served as a control, that did not show any alterations during the ophthalmological evaluation or any vitreoretinal and vascular retinal diseases.

Each subject underwent a complete ophthalmological evaluation including the measurement of best-corrected visual acuity (BCVA) according to the Early Treatment of Diabetic Retinopathy Study (ETDRS) [9], slit-lamp biomicroscopy, fundus examination, intraocular pressure, evaluation of the SD-OCT parameters (ganglion cell complex, retinal nerve fiber layer) and OCTA parameters. The BCVA was converted into a logarithm of the minimum angle of resolution (log MAR) scale for statistical calculations.

SD-OCT and OCTA measurements were performed by two examiners (DM, LD) and a senior expert (GC) that checked the quality of SD-OCT and OCTA images.

The study adhered to the tenets of the Declaration of Helsinki. Written informed consent was obtained from the patients enrolled in the study. The research protocol was registered on Clinical Trials.gov (NCT04546997).

### 2.2. Spectral Domain Optical Coherence Tomography

After pupillary dilation (minimum diameter 5 mm), we evaluated the mean circumpapillary RNFL and GCC thickness with SD-OCT (software RTVue XR version 2017.1.0.151, Optovue Inc., Fremont, CA, USA) which captures 26,000 axial scans (A-scans) per second and provides a 5 µm depth resolution in tissue. The circumpapillary RNFL was evaluated by the optic nerve head (ONH) map protocol, and RNFL thickness measurements were obtained using a 3.45 mm radius ring centred on the optic disc. The GCC scan data were displayed as a thickness map of the GCC layer. The device measures GCC thickness within an automatically rendered 7 mm^2^ area, centred 1 mm temporal to the fovea. The GCC thickness was analysed from the internal limiting membrane to the outer boundary of the inner plexiform layer.

We accepted only scans with high image quality (signal strength index (SSI) > 40). The measurements of GCC thickness were compared with values from a normative database. The device software then produced a colour-coded GCC thickness map [10].

Scans were rejected if they were of poor quality, had a signal strength index (SSI) less than 40, or demonstrated incorrect segmentation, motion artefacts or low centration.

### 2.3. Optical Coherence Tomography Angiography

OCTA was performed by the Optovue Angiovue System (software ReVue XR version 2017.1.0.151, Optovue Inc., Fremont, CA, USA) that is based on a split-spectrum amplitude de-correlation algorithm (SSADA) and which uses blood flow as intrinsic contrast [11].

Measurements of the macular capillary network were performed in a 6×6 mm scan centred on the fovea. The OCTA software analysed the macular region divided in whole image, fovea and parafovea in each vascular network of the retina: superficial capillary plexus (SCP) and deep capillary plexus (DCP), according to the ETDRS classification of diabetic retinopathy.

The SCP was automatically selected at a 60 μm thickness from the inner limiting membrane (ILM) to include all the vessels of this plexus. A 30-μm-thick layer from the inner plexiform layer visualised the entire DCP automatically.

In each retinal vascular network, the AngioAnalytics™ software (Optovue Inc., Fremont, CA, USA) automatically calculated the vessel density (VD) that was defined as the proportion of vessel area with flowing blood over the total measurement area [12]. The AngioVue disc mode automatically calculated the VD of the radial peripapillary capillary plexus (RPC). We analysed the VD in the superficial retinal layers and extended from the ILM to the RNFL posterior boundary. The VD measurements of the RPC were performed over a scan area of 4.5×4.5 mm centred on the optic disc (whole image), inside the optic disc and in the peripapillary region that extends in a 0.75-mm-wide elliptical annulus from the ONH boundary [13]. AngioVue software automatically calculated the foveal avascular zone (FAZ) area in square millimetres over the 6x6 mm macular area in the full retinal plexus [14]. Images with a signal strength index less than 40 or residual motion artefacts, incorrect segmentation or low centration and focus were excluded.

### 2.4. Statistical Analysis

The Statistical Package for Social Sciences (Version 25 for Windows; SPSS Inc, Chicago, IL, USA) was used for statistical analyses. The Shapiro–Wilk test confirmed that all variables were normally distributed. Data were analysed by one-way analysis of variance (ANOVA) followed by Bonferroni post hoc analysis. We determined the differences in structural SD-OCT and OCTA parameters of each ocular trauma eye and their healthy control eyes. A *p* value of <0.05 was considered statistically significant.

## 3. Results

A total of 18 eyes of 18 patients (8 males, 10 females, mean age 49.61 ± 9.2 years) with a previous episode of blunt ocular trauma were enrolled in this prospective study.

At 48 h after the trauma, the patients showed a slight, but not statistically significant, decrease in BCVA compared to the control group (0.07 ± 0.09 logMAR vs. 0.12 ± 0.11 logMAR; *p* = 0.820). At 1, 3 and 6 months, the BCVA values did not significantly differ from those of the control group.

The structural SD-OCT examination showed no statistically significant differences in GCC parameters between the eyes evaluated at 48 h after trauma and the controls (*p* = 0.984). At 1, 3 and 6 months, the GCC of traumatised eyes was significantly thinner than that of the control group (*p* = 0.021, *p* < 0.001, *p* < 0.001). The GCC thickness did not change at 6 months in respect to the third month (*p* = 0.859). Unlike GCC, the RNFL showed a decrease at 3 and 6 months in respect to controls (*p* < 0.001) (Table 1).

A similar trend was found in OCTA parameters that showed no significant differences in the VD of the SCP in different macular regions between the control group and the eyes affected by trauma at 48 h follow-up (*p* = 0.912). The VD of the SCP showed progressive and significant reduction in respect to controls at 1 and 3 months (*p* = 0.013, *p* < 0.001). At 6 months, the VD values did not change compared to the third month (*p* = 0.985).

**Figure 1:**
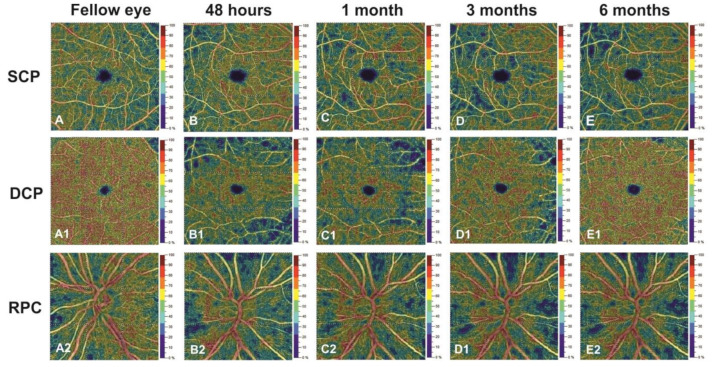
Right eye of a 48-year-old patient evaluated at 48 h (**B**,**B1**,**B2**), 1 month **(C**,**C1**,**C2**), 3 months (**D**,**D1**,**D2**) and 6 months (**E**,**E1**,**E2**) after ocular trauma compared to healthy fellow eye (**A**,**A1**,**A2**), using optical coherence tomography angiography. Compared to the fellow (control) eye, the superficial capillary plexus (**B**,**C**,**D**,**E**) and radial peripapillary capillary (RPC) (**B2**,**C2**,**D2**,**E2**) showed a progressive focal reduction in vessel density (VD) until the third month. The VD values stabilised in the sixth month. The deep capillary plexus revealed, after an initial reduction at 48 h, a progressive increase in the VD in respect to the fellow eye until the third month, and it reached the plateau at 6 months (**B1**,**C1**,**D1**,**E1**).

The papillary region displayed a significant decrease in the VD at 3 and 6 months after the trauma compared to controls (*p* < 0.001). No significant difference was shown at 6 months in respect to VD values in the third month (*p* = 0.988). Different behaviour was shown in the DCP in all macular sectors that presented a statistically significant decrease in VD at 48 h compared to the control group (*p* < 0.001). Furthermore, the VD values statistically increased in the first month of follow-up. At 3 and 6 months, the values of DCP were similar to those of the control group (*p* > 0.05). The FAZ area did not change during the 6 months follow-up (*p* > 0.05) (Table 2, Figure 1).

## 4. Discussion

In this prospective study, we aimed to analyse the structural and retinal vascular changes in macular and papillary regions over a 6 months follow-up period after blunt ocular trauma.

We found, at only 1 month after the commotio retinae, an initial significant reduction in GCC. This result highlights the role of the retinal ganglion cells as the initial retinal parameter sensitive to early changes due to traumatic insult [5]. During the follow-up, the structural OCT parameters progressively decreased until they stabilised at the sixth month, confirming previous studies that reported a significant impairment of the RGCs and their axons starting at 2 weeks and plateauing at 20 weeks follow-up [5,6,7].

Recently, OCTA has proved to be a very useful and non-invasive diagnostic tool, allowing for the investigation of the possible vascular involvement in retinal structural changes occurring after ocular trauma.

We found a statistically significant decrease in SCP at the first month, despite the preserved visual acuity, supporting the hypothesis that retinal microvascular dysfunction could occur even in the absence of functional loss.

After the third month post-trauma, the papillary vascular network and the RNFL showed a progressive impairment. The structural loss together with vascular damage was confirmed by the fact that the superficial retinal vascular network and the radial peripapillary vascular plexus are located, respectively, in the ganglion cell layer and the peripapillary RNFL, and they are responsible for their metabolic demand [15].

Our findings could be explained by a possible mechanical and hemodynamic pathogenesis of the commotio retinae [4]. The neurosensory retina may be stretched by the direct mechanical forces at the level of the vulnerable ellipsoid and interdigitation zones, leading to outer retinal alterations. This force could also be transmitted to the retinal microvascularisation, causing reduced blood flow [4]. These microvascular alterations, following the trauma, would demonstrate the impaired vascular perfusion of the SCP and the RPC that could have caused local ischaemia at the level of the optic nerve resulting in interference in axoplasmic flow and subsequent retinal structural loss [16]. The hemodynamic pathogenesis of the ocular trauma could also explain the early decreased VD of the DCP because it was vulnerable to ischaemic insult due to its reduced vascular calibre [15].

Our results indicated a significant improvement of the DCP in patients that did not present any reduction in visual acuity in the 6 months of follow-up, confirming the role of this retinal vascular network in the metabolic demand of the outer retinal and, in particular, the photoreceptors layer and RPE [17]. Therefore, our findings showed that, although the functional outcomes were not significantly compromised, the retinal microvascular changes occurred in early phases after ocular trauma.

Few studies have reported the analysis of the retinal vascular network by OCTA. Shields et al. showed no microvascular alteration in superficial capillary density and FAZ in a case of a man after tennis ball injury, probably due to fact that the exam was performed 24 h after trauma and it lacked the phase of vascular spasm choroidal vascularisation [18].

Wangsathaporn et al. revealed no retinal perfusion defects in two patients affected by commotio retinae following rubber band injuries, despite OCT performed after 1 year of persistent defects of the outer retinal layer [19].

Different results were shown by Papageorgiou, in which three cases of commotio retinae in paediatric patients showed an enlargement of the FAZ twelve hours after injury and, in severe cases, also a reduction of the superficial foveal capillary density after eleven months [20].

Our study demonstrated the progressive increase of the VD of DCP that could contribute to restoring the outer retinal structural features after trauma, confirming the role of the retinal deep vascular network in the metabolic demand of the outer retinal layers [17].

We hypothesised that the early damage in DCP after trauma could be due to its anatomical vascular structure characterised by a fine capillary network that is, therefore, more sensitive to non-perfusion damage in respect to SCP and RPC that show a greater vascular calibre. Reduced blood flow could explain the impairment of the retinal vessel density due to the mechanical effect of the trauma rather than the presence of the tissue oedema in the macular region. These findings are confirmed by previous histological studies that found structural disorganisation of the outer retinal layers without intraretinal fluid [2,21,22].

The main limitations of this study are represented by the small sample size and the absence of a longer follow-up. In conclusion, in this prospective study, OCTA and SD-OCT provided useful information regarding the early changes in the different retinal vascular networks and retinal structural parameters after commotio retinae. Longitudinal studies on larger cohorts, also involving the choroidal structure, are needed to investigate the role of early vascular alterations during the progression of structural changes after ocular trauma.

## Figures and Tables

**Table 1:** Differences in structural SD-OCT (spectral-domain optical coherence tomography) parameters among the patients evaluated at each control after ocular trauma and their healthy fellow eyes.

	Control Group	48 Hours	1 Month	3 Months	6 Months	ANOVA
						*p*
**GCC (µm)**						
**Average**	105.55 ± 7.45	105.38 ± 7.56	94.55 ± 6.38	88.05 ± 5.76	88.11 ± 5.76	<0.001
**Superior**	106.66 ± 7.95	106.38 ± 7.94	94.66 ± 5.43	90.61 ± 3.03	90.72 ± 3.04	<0.001
**Inferior**	104.16 ± 7.83	104.05 ± 7.99	94.22 ± 6.69	86.38 ± 4.27	86.50 ± 4.28	<0.001
**RNFL (µm)**						
**Average**	110.44 ± 4.74	110.55 ± 5.54	109.50 ± 5.61	98.33 ± 3.80	98.61 ± 5.58	<0.001
**Superior**	112.45 ± 6.59	111.46 ± 5.78	110.88 ± 6.07	100.27 ± 5.73	99.05 ± 3.42	<0.001
**Inferior**	108.50 ± 4.66	106.27 ± 4.86	105.77 ± 5.09	96.38 ± 6.16	99.72 ± 5.73	<0.001

Data are expressed as mean ± SD; GCC: ganglion cell complex; RNFL: retinal nerve Fiber layer; one-way analysis of variance (ANOVA) followed by Bonferroni post hoc analysis, statistical significance *p* value < 0.05.

**Table 2:** Differences in OCT angiography parameters among the patients evaluated at each control after ocular trauma and their healthy fellow eyes.

	Control Group	48 Hours	1 Month	3 Months	6 Months	ANOVA
						*p*
**SCP (%)**						
**Whole Image**	51.38 ± 4.47	51.45 ± 4.28	46.12 ± 5.95	44.45 ± 4.65	44.23 ± 4.09	<0.001
**Parafovea**	53.77 ± 4.58	52.61 ± 4.59	47.50 ± 6.01	46.55 ± 4.67	46.11 ± 5.36	<0.001
**Fovea**	27.94 ± 4.33	26.55 ± 4.35	23.35 ± 4.29	22.53 ± 4.53	22.12 ± 5.12	<0.001
**DCP (%)**						
**Whole Image**	55.72 ± 5.22	48.33 ± 5.17	54.38 ± 4.23	55.16 ± 4.55	55.50 ± 4.41	<0.001
**Parafovea**	58.55 ± 3.46	53.23 ± 4.53	57.95 ± 5.90	58.06 ± 4.15	58.23 ± 5.32	0.005
**Fovea**	43.16 ± 2.97	35.21 ± 3.32	41.26 ± 6.54	41.16 ± 5.99	42.82 ± 3.67	<0.001
**FAZ (mm^2^)**	0.310 ± 0.14	0.308 ± 0.15	0.311 ± 0.18	0.309 ± 0.12	0.307 ± 0.10	0.998
**RPC (%)**						
**Whole Image**	55.55 ± 4.09	55.21 ± 6.09	54.08 ± 3.98	46.31 ± 3.72	46.13 ± 5.01	<0.001
**Inside Disc**	51.88 ± 3.49	50.19 ± 4.68	50.20 ± 4.21	44.86 ± 3.90	43.88 ± 3.18	<0.001
**Peripapillary Region**	54.44 ± 3.11	54.31 ± 3.71	53.52 ± 3.77	45.75 ± 3.85	45.75 ± 4.16	<0.001

Data are expressed as mean ± SD; SCP: superficial capillary plexus; DCP: deep capillary plexus; FAZ: foveal avascular zone; RPC: radial peripapillary capillary plexus. One-way analysis of variance (ANOVA) followed by Bonferroni post hoc analysis, statistical significance *p* value < 0.05.
